# Complete Genome Sequence of Bacillus mycoides TH26, Isolated from a Permafrost Thaw Gradient

**DOI:** 10.1128/MRA.00507-19

**Published:** 2019-06-13

**Authors:** Tracie Haan, Scout McDougall, Devin M. Drown

**Affiliations:** a Department of Biology and Wildlife, University of Alaska Fairbanks, Fairbanks, Alaska, USA; b Institute of Arctic Biology, University of Alaska Fairbanks, Fairbanks, Alaska, USA; Loyola University Chicago

## Abstract

Here, we describe the complete genome assembly of a Bacillus mycoides isolate collected from a boreal forest soil associated with permafrost thaw. Using only long-read Nanopore sequences and VolTRAX library preparation, we assembled two circular contigs totaling 5,789,722 bp (*N*_50_, 5,306,036), a complete chromosome with one plasmid.

## ANNOUNCEMENT

We collected soil from the Fairbanks Permafrost Experiment Station in Alaska (64.875646°N, 147.668981°W). We have been sampling at this site to understand how belowground microbial communities mediate the effect of permafrost thaw on aboveground plant communities in boreal and arctic ecosystems. The psychrotolerant Bacillus mycoides isolate described here comes from an undergraduate-driven project on the coselection of heavy metal tolerance and antibiotic resistance, and it is associated with a permafrost thaw feature.

We homogenized the active layer from two soil cores and added 1 g of homogenized sample to tryptic soy broth (TSB). After 48 h at 22°C, we plated a dilution on tryptic soy agar and used the streak plate method three times to purify our randomly selected isolate. From this purified isolate, we inoculated a liquid culture of TSB, incubated it at 22°C overnight, and used 1.8 ml to extract DNA using the DNeasy UltraClean microbial kit (Qiagen).

We used 1 μg of DNA as input for the VolTRAX V2 (Oxford Nanopore Technologies [ONT]) to prepare a sequencing library (VSK-VSK002 workflow). We sequenced the prepared library using a MinION device (ONT) and an r9.4.1 flow cell (FLO-MIN106) for 48 h (VSK002 script). We base called the raw data using Guppy v2.3.1 (ONT), specifying the flipflop model (–c dna_r9.4.1_450bps_flipflop.cfg) and default parameters (Table 1). Before assembly, we created a quality-controlled data set using Porechop v0.2.4 (https://github.com/rrwick/Porechop) with default parameters to trim adaptors and discard sequences with middle adapters (-discard_middle) and Filtlong v0.2.0 (https://github.com/rrwick/Filtlong) to filter by lengths of ≥1,000 bp (-min_length 1000) and quality (*Q* score) of ≥10 (-min_mean_q 90).

**TABLE 1:** Summary of sequencing data statistics

Nanopore data set	No. of reads	Yield (bp)	Avg length (bp)	Avg quality (*Q* score)
Base called	4,307,730	14,088,429,685	3,270	11.4
Quality controlled	2,990,198	11,360,386,781	3,799	12.5
Subsample	79,242	1,000,003,941	12,619	12.7

Before genome assembly, we subsampled 1 Gb of the quality-controlled data using Filtlong (-length_weight 10 -target_bases 1000000000) and used the longer reads for assembly. We assembled the genome using Canu v1.8 (1) (coverage, 200×) with default parameters, specifying the estimated genome size (-genomeSize=5m) and Nanopore reads (-nanopore-raw). Before using medaka (https://github.com/nanoporetech/medaka) for polishing, ONT recommends 4 rounds of Racon v1.3.1 (2) polishing with the following parameters: score for matching bases (-match 8), score for mismatching bases (-mismatch -6), threshold for average base quality of windows (-quality-threshold -1), default gap penalty (-gap -8), and default window (-window-length 500). We ran a final polish with medaka v0.6.1, specifying the flipflop model (-m r941_flip) and default parameters. For both Racon and medaka polishing, we used the entire 11-Gb quality-controlled data set (coverage, 1,958×).

Our 5,789,722-bp (*N*_50_, 5,306,036) polished assembly consists of 2 circular contigs, a 5,306,036-bp chromosome (GC content, 35.57%), and a 483,686-bp plasmid (GC content, 33.56%). We validated our assembly at multiple checkpoints using CheckM v1.0.11 (3) in the following lineage-specific workflow (lineage_wf): before polishing (completeness, 83.14%; contamination, 0.15%), after initial polishing with Racon (completeness, 94.67%; contamination, 0.07%), and after final Medaka polishing (completeness 98.21%; contamination, 0.15%).

We used PATRIC v3.5.36 (4) to assign 107 tRNAs, 28 rRNAs, and 6,458 coding sequences (CDS). PATRIC assigned the isolate as a member of the Bacillus cereus group, with close identity to the psychrotolerant species Bacillus mycoides. To assign taxonomy, we used blastn (5) to search against the NCBI 16S rRNA database and found 100% identity to Bacillus mycoides (strain DSM 11821). Therefore, we assign this isolate as Bacillus mycoides TH26.

### Data availability.

This whole-genome shotgun project has been deposited in GenBank under the accession no. CP037991 and CP037992. The versions described in this paper are the first versions, CP037991.1 and CP037992.1. Raw data for this project can be found in the GenBank SRA under accession no. PRJNA525875.
